# 4-Chloro-2-{3-chloro-2-[(3,5-dimethyl­piperidin-1-yl)meth­yl]phenyl­sulfan­yl}-6-methoxy­pyrimidine

**DOI:** 10.1107/S1600536810004599

**Published:** 2010-02-17

**Authors:** Guanglong Zou, Mei Zhu

**Affiliations:** a School of Chemistry and Environmental Science, Guizhou University for Nationalities, Guiyang 550025, People’s Republic of China; b College of Chemistry and Chemical Engineering, Luoyang Normal University, Luoyang 471022, People’s Republic of China

## Abstract

In the title compound, C_19_H_23_Cl_2_N_3_OS, the dihedral angle between the benzene ring and the pyrimidine ring is 86.6 (9)°. The piperidine ring adopts a chair conformation.

## Related literature

For the biological activity of pyrimidine derivatives, see: Joffe *et al.* (1989 ▶); Petersen & Schmidt (2003 ▶); Blum (2001 ▶); Gompper *et al.* (2004 ▶); Michael (2005 ▶); Nadal & Olavarria (2004 ▶).
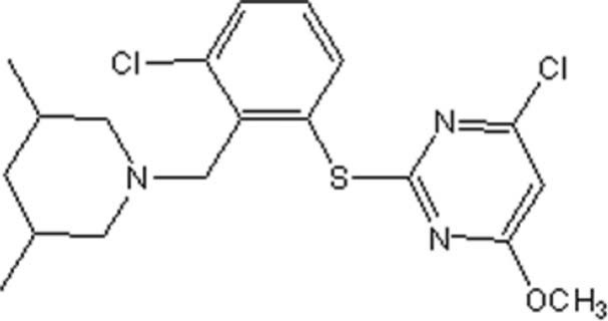


## Experimental

### 

#### Crystal data

C_19_H_23_Cl_2_N_3_OS
*M* *_r_* = 412.36
Triclinic, 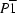

*a* = 8.000 (4) Å
*b* = 11.454 (6) Å
*c* = 12.001 (7) Å
α = 87.820 (7)°
β = 76.084 (6)°
γ = 77.700 (6)°
*V* = 1042.9 (10) Å^3^
*Z* = 2
Mo *K*α radiation
μ = 0.42 mm^−1^
*T* = 296 K
0.39 × 0.37 × 0.25 mm

#### Data collection

Bruker SMART APEXII CCD area-detector diffractometer
Absorption correction: multi-scan (*SADABS*; Sheldrick, 1996 ▶) *T*_min_ = 0.852, *T*_max_ = 0.901
7861 measured reflections
3848 independent reflections
2553 reflections with *I* > 2σ(*I*)
*R*_int_ = 0.019

#### Refinement

*R*[*F* ^2^ > 2σ(*F* ^2^)] = 0.041
*wR*(*F* ^2^) = 0.119
*S* = 1.03
3848 reflections
238 parameters
H-atom parameters constrained
Δρ_max_ = 0.30 e Å^−3^
Δρ_min_ = −0.31 e Å^−3^

### 

Data collection: *APEX2* (Bruker, 2004 ▶); cell refinement: *SAINT* (Bruker, 2004 ▶); data reduction: *SAINT*; program(s) used to solve structure: *SHELXS97* (Sheldrick, 2008 ▶); program(s) used to refine structure: *SHELXL97* (Sheldrick, 2008 ▶); molecular graphics: *SHELXTL* (Sheldrick, 2008 ▶); software used to prepare material for publication: *SHELXTL* and *PLATON* (Spek, 2009 ▶).

## Supplementary Material

Crystal structure: contains datablocks global, I. DOI: 10.1107/S1600536810004599/pb2021sup1.cif

Structure factors: contains datablocks I. DOI: 10.1107/S1600536810004599/pb2021Isup2.hkl

Additional supplementary materials:  crystallographic information; 3D view; checkCIF report

## Figures and Tables

**Fig. 1.**
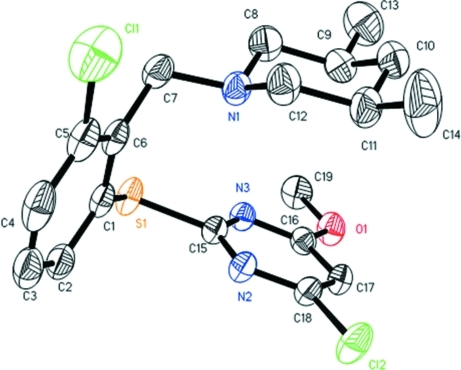
View of the molecular structure of (I) with atom numbering scheme and 30% probability displacement ellipsoids.

## supplementary crystallographic information

### Comment

Pyrimidine derivatives are widespread in medicinal and natural products chemistry. A number of natural products, pharmaceuticals, and functional materials incorporate this heterocycle (Michael, 2005. Several examples of pharmaceutically important compounds include trimethoprim (Joffe *et al.*, 1989), sulfadiazine (Petersen & Schmidt, 2003),Gleevec (imatinib mesilate) (Nadal & Olavarria, 2004), and Xeloda (capecitabine) (Blum, 2001). Natural and unnatural polymers also contain pyrimidine derivatives (Gompper *et al.*, 2004). The potent physiological properties of these pyrimidine derivatives led to vast research of their use as medicines in the field of pharmaceutical chemistry. So in the recent decades, many chemists have been attracted by the synthesis of pyrimidines. In this context, we report the synthesis ofthe title compound.

The molecular structure is shown in Fig. 1. The bond lengths and angles are within normal ranges. The pyrimidine ring makes dihedral angles of 86.6 (9)°  with the benzene ring. In the crystal structure,The cyclohexyl groups display chair-type conformation.

### Experimental

To a solution of 2,4-dichloro-6-methoxypyrimidine (0.5 mmol) and 3-chloro-2-((3,5-dimethylpiperidin-1-yl)methyl)benzenethiol (0.5 mmol) in dry methylbenzene was added NaH (0.6 mmol). The mixture was stirred for 12 h at room temperature. After evaporation of the solvent, the residue was purified by column chromatography on silica gel to afford the title compound as a colorless solid (yield 78%). The title compound was recrystallized from CH_2_Cl_2_ at room temperature to give the desired crystals suitable for single-crystal X-ray diffraction.

### Refinement

All H atoms were positioned geometrically and treated as riding, with C—H bond lengths constrained to 0.93 Å (aromatic CH) or 0.97 Å (methylene CH_2_), and with *U*ĩso~(H) = 1.2Ueq(C) or 1.5Ueq(methylene C).

### Crystal data

**Table:**

C_19_H_23_Cl_2_N_3_OS	*Z* = 2
*M**_r_* = 412.36	*F*(000) = 432
Triclinic, *P*1	*D*_x_ = 1.313 Mg m^−^^3^
*a* = 8.000 (4) Å	Mo *K*α radiation, λ = 0.71073 Å
*b* = 11.454 (6) Å	Cell parameters from 2557 reflections
*c* = 12.001 (7) Å	θ = 2.5–25.9°
α = 87.820 (7)°	µ = 0.42 mm^−^^1^
β = 76.084 (6)°	*T* = 296 K
γ = 77.700 (6)°	Block, colourless
*V* = 1042.9 (10) Å^3^	0.39 × 0.37 × 0.25 mm

### Data collection

**Table:**

Bruker SMART APEXII CCD area-detector diffractometer	3848 independent reflections
Radiation source: fine-focus sealed tube	2553 reflections with *I* > 2σ(*I*)
graphite	*R*_int_ = 0.019
phi and ω scans	θ_max_ = 25.5°, θ_min_ = 2.5°
Absorption correction: multi-scan (*SADABS*; Sheldrick, 1996)	*h* = −9→9
*T*_min_ = 0.852, *T*_max_ = 0.901	*k* = −13→13
7861 measured reflections	*l* = −14→14

### Refinement

**Table:**

Refinement on *F*^2^	Primary atom site location: structure-invariant direct methods
Least-squares matrix: full	Secondary atom site location: difference Fourier map
*R*[*F*^2^ > 2σ(*F*^2^)] = 0.041	Hydrogen site location: inferred from neighbouring sites
*wR*(*F*^2^) = 0.119	H-atom parameters constrained
*S* = 1.02	*w* = 1/[σ^2^(*F*_o_^2^) + (0.0498*P*)^2^ + 0.3748*P*] where *P* = (*F*_o_^2^ + 2*F*_c_^2^)/3
3848 reflections	(Δ/σ)_max_ < 0.001
238 parameters	Δρ_max_ = 0.30 e Å^−^^3^
0 restraints	Δρ_min_ = −0.30 e Å^−^^3^

### Special details

**Table:**

Geometry. All esds (except the esd in the dihedral angle between two l.s. planes) are estimated using the full covariance matrix. The cell esds are taken into account individually in the estimation of esds in distances, angles and torsion angles; correlations between esds in cell parameters are only used when they are defined by crystal symmetry. An approximate (isotropic) treatment of cell esds is used for estimating esds involving l.s. planes.
Refinement. Refinement of F^2^ against ALL reflections. The weighted R-factor wR and goodness of fit S are based on F^2^, conventional R-factors R are based on F, with F set to zero for negative F^2^. The threshold expression of F^2^ > 2sigma(F^2^) is used only for calculating R-factors(gt) etc. and is not relevant to the choice of reflections for refinement. R-factors based on F^2^ are statistically about twice as large as those based on F, and R- factors based on ALL data will be even larger.

### Fractional atomic coordinates and isotropic or equivalent isotropic displacement parameters (Å^2^)

**Table:**

	*x*	*y*	*z*	*U*_iso_*/*U*_eq_	
C1	0.8993 (3)	0.4679 (2)	0.3790 (2)	0.0511 (6)	
C2	0.7914 (4)	0.5783 (3)	0.4072 (2)	0.0639 (7)	
H2	0.8031	0.6417	0.3572	0.077*	
C3	0.6657 (4)	0.5951 (3)	0.5096 (3)	0.0772 (10)	
H3	0.5913	0.6695	0.5281	0.093*	
C4	0.6503 (4)	0.5026 (4)	0.5841 (3)	0.0765 (10)	
H4	0.5657	0.5136	0.6533	0.092*	
C5	0.7618 (4)	0.3926 (3)	0.5555 (2)	0.0649 (8)	
C6	0.8897 (3)	0.3706 (2)	0.4527 (2)	0.0517 (6)	
C7	1.0146 (4)	0.2522 (3)	0.4215 (2)	0.0613 (7)	
H7A	1.1342	0.2648	0.3977	0.074*	
H7B	1.0071	0.2019	0.4886	0.074*	
C8	1.1282 (4)	0.1048 (3)	0.2685 (3)	0.0722 (8)	
H8A	1.1601	0.0419	0.3206	0.087*	
H8B	1.2269	0.1439	0.2432	0.087*	
C9	1.0929 (4)	0.0498 (3)	0.1644 (3)	0.0773 (9)	
H9	1.0612	0.1145	0.1126	0.093*	
C10	0.9343 (4)	−0.0090 (3)	0.2069 (3)	0.0838 (10)	
H10A	0.9048	−0.0403	0.1418	0.101*	
H10B	0.9647	−0.0753	0.2562	0.101*	
C11	0.7761 (4)	0.0801 (3)	0.2730 (3)	0.0867 (10)	
H11	0.7438	0.1436	0.2202	0.104*	
C12	0.8245 (4)	0.1367 (3)	0.3699 (3)	0.0757 (9)	
H12A	0.7247	0.1972	0.4083	0.091*	
H12B	0.8503	0.0761	0.4256	0.091*	
C13	1.2537 (5)	−0.0357 (3)	0.1000 (3)	0.1102 (13)	
H13A	1.3485	0.0055	0.0749	0.165*	
H13B	1.2284	−0.0676	0.0345	0.165*	
H13C	1.2871	−0.0998	0.1494	0.165*	
C14	0.6166 (5)	0.0238 (4)	0.3187 (5)	0.151 (2)	
H14A	0.6428	−0.0360	0.3738	0.226*	
H14B	0.5894	−0.0124	0.2562	0.226*	
H14C	0.5174	0.0844	0.3547	0.226*	
C15	0.9754 (3)	0.4023 (2)	0.14901 (19)	0.0466 (6)	
C16	1.0372 (3)	0.3253 (2)	−0.0292 (2)	0.0483 (6)	
C17	0.8596 (3)	0.3280 (2)	−0.0188 (2)	0.0528 (6)	
H17	0.8179	0.3030	−0.0778	0.063*	
C18	0.7513 (3)	0.3697 (2)	0.0833 (2)	0.0500 (6)	
C19	1.3329 (4)	0.2760 (3)	−0.1362 (3)	0.0729 (8)	
H19A	1.3525	0.3554	−0.1317	0.109*	
H19B	1.3998	0.2398	−0.2085	0.109*	
H19C	1.3695	0.2292	−0.0749	0.109*	
Cl1	0.73889 (14)	0.27940 (11)	0.65587 (8)	0.1117 (4)	
Cl2	0.52759 (9)	0.37327 (8)	0.10766 (6)	0.0780 (3)	
N1	0.9758 (3)	0.19101 (19)	0.32894 (19)	0.0585 (6)	
N2	0.8031 (3)	0.40866 (18)	0.16994 (16)	0.0497 (5)	
N3	1.0978 (3)	0.36245 (17)	0.05379 (16)	0.0480 (5)	
O1	1.1505 (2)	0.28158 (17)	−0.12653 (14)	0.0651 (5)	
S1	1.06955 (9)	0.45367 (7)	0.25092 (6)	0.0613 (2)	

### Atomic displacement parameters (Å^2^)

**Table:**

	*U*^11^	*U*^22^	*U*^33^	*U*^12^	*U*^13^	*U*^23^
C1	0.0428 (14)	0.0719 (18)	0.0443 (13)	−0.0167 (13)	−0.0152 (11)	−0.0130 (13)
C2	0.0580 (18)	0.0735 (19)	0.0658 (18)	−0.0100 (15)	−0.0263 (15)	−0.0174 (15)
C3	0.0500 (18)	0.095 (2)	0.088 (2)	−0.0002 (16)	−0.0258 (17)	−0.043 (2)
C4	0.0440 (17)	0.127 (3)	0.0581 (18)	−0.0211 (19)	−0.0025 (13)	−0.0381 (19)
C5	0.0497 (16)	0.102 (2)	0.0489 (15)	−0.0256 (16)	−0.0123 (12)	−0.0129 (15)
C6	0.0387 (14)	0.0765 (18)	0.0458 (14)	−0.0172 (13)	−0.0144 (11)	−0.0118 (13)
C7	0.0502 (16)	0.0762 (19)	0.0630 (17)	−0.0127 (14)	−0.0235 (13)	−0.0044 (14)
C8	0.0544 (18)	0.075 (2)	0.086 (2)	−0.0077 (15)	−0.0158 (15)	−0.0109 (16)
C9	0.088 (2)	0.0613 (19)	0.084 (2)	−0.0076 (17)	−0.0285 (18)	−0.0092 (16)
C10	0.090 (2)	0.063 (2)	0.104 (3)	−0.0093 (18)	−0.038 (2)	−0.0161 (18)
C11	0.069 (2)	0.078 (2)	0.126 (3)	−0.0152 (17)	−0.044 (2)	−0.019 (2)
C12	0.0523 (18)	0.080 (2)	0.096 (2)	−0.0149 (15)	−0.0165 (16)	−0.0183 (17)
C13	0.120 (3)	0.099 (3)	0.097 (3)	−0.015 (2)	−0.003 (2)	−0.023 (2)
C14	0.081 (3)	0.149 (4)	0.234 (6)	−0.048 (3)	−0.028 (3)	−0.070 (4)
C15	0.0469 (15)	0.0525 (15)	0.0424 (13)	−0.0118 (12)	−0.0128 (11)	−0.0016 (11)
C16	0.0533 (16)	0.0477 (14)	0.0413 (13)	−0.0097 (11)	−0.0065 (11)	−0.0045 (11)
C17	0.0575 (16)	0.0600 (16)	0.0461 (14)	−0.0163 (13)	−0.0179 (12)	−0.0061 (12)
C18	0.0462 (15)	0.0567 (15)	0.0498 (14)	−0.0110 (12)	−0.0159 (12)	−0.0012 (12)
C19	0.0481 (17)	0.087 (2)	0.0704 (19)	−0.0035 (15)	0.0056 (14)	−0.0227 (16)
Cl1	0.1161 (8)	0.1555 (10)	0.0669 (5)	−0.0582 (7)	−0.0048 (5)	0.0201 (6)
Cl2	0.0450 (4)	0.1197 (7)	0.0740 (5)	−0.0198 (4)	−0.0189 (3)	−0.0135 (4)
N1	0.0447 (13)	0.0622 (14)	0.0712 (14)	−0.0079 (11)	−0.0197 (11)	−0.0128 (11)
N2	0.0428 (12)	0.0640 (13)	0.0436 (11)	−0.0108 (10)	−0.0120 (9)	−0.0063 (9)
N3	0.0459 (12)	0.0536 (12)	0.0436 (11)	−0.0111 (9)	−0.0076 (9)	−0.0041 (9)
O1	0.0607 (12)	0.0770 (13)	0.0517 (11)	−0.0109 (10)	−0.0021 (9)	−0.0198 (9)
S1	0.0499 (4)	0.0947 (6)	0.0468 (4)	−0.0301 (4)	−0.0108 (3)	−0.0118 (3)

### Geometric parameters (Å, °)

**Table:**

C1—C2	1.374 (4)	C11—C14	1.527 (5)
C1—C6	1.400 (4)	C11—H11	0.9800
C1—S1	1.778 (3)	C12—N1	1.451 (3)
C2—C3	1.378 (4)	C12—H12A	0.9700
C2—H2	0.9300	C12—H12B	0.9700
C3—C4	1.367 (5)	C13—H13A	0.9600
C3—H3	0.9300	C13—H13B	0.9600
C4—C5	1.381 (4)	C13—H13C	0.9600
C4—H4	0.9300	C14—H14A	0.9600
C5—C6	1.392 (4)	C14—H14B	0.9600
C5—Cl1	1.744 (3)	C14—H14C	0.9600
C6—C7	1.504 (4)	C15—N2	1.327 (3)
C7—N1	1.462 (3)	C15—N3	1.336 (3)
C7—H7A	0.9700	C15—S1	1.759 (2)
C7—H7B	0.9700	C16—N3	1.327 (3)
C8—N1	1.450 (3)	C16—O1	1.334 (3)
C8—C9	1.531 (4)	C16—C17	1.390 (3)
C8—H8A	0.9700	C17—C18	1.356 (3)
C8—H8B	0.9700	C17—H17	0.9300
C9—C13	1.501 (5)	C18—N2	1.331 (3)
C9—C10	1.533 (4)	C18—Cl2	1.735 (3)
C9—H9	0.9800	C19—O1	1.423 (3)
C10—C11	1.517 (4)	C19—H19A	0.9600
C10—H10A	0.9700	C19—H19B	0.9600
C10—H10B	0.9700	C19—H19C	0.9600
C11—C12	1.518 (4)		
			
C2—C1—C6	122.1 (2)	C10—C11—H11	107.8
C2—C1—S1	118.1 (2)	C14—C11—H11	107.8
C6—C1—S1	119.5 (2)	N1—C12—C11	112.1 (3)
C1—C2—C3	120.0 (3)	N1—C12—H12A	109.2
C1—C2—H2	120.0	C11—C12—H12A	109.2
C3—C2—H2	120.0	N1—C12—H12B	109.2
C4—C3—C2	120.1 (3)	C11—C12—H12B	109.2
C4—C3—H3	119.9	H12A—C12—H12B	107.9
C2—C3—H3	119.9	C9—C13—H13A	109.5
C3—C4—C5	119.2 (3)	C9—C13—H13B	109.5
C3—C4—H4	120.4	H13A—C13—H13B	109.5
C5—C4—H4	120.4	C9—C13—H13C	109.5
C4—C5—C6	122.9 (3)	H13A—C13—H13C	109.5
C4—C5—Cl1	116.9 (2)	H13B—C13—H13C	109.5
C6—C5—Cl1	120.1 (2)	C11—C14—H14A	109.5
C5—C6—C1	115.7 (3)	C11—C14—H14B	109.5
C5—C6—C7	123.5 (3)	H14A—C14—H14B	109.5
C1—C6—C7	120.8 (2)	C11—C14—H14C	109.5
N1—C7—C6	112.1 (2)	H14A—C14—H14C	109.5
N1—C7—H7A	109.2	H14B—C14—H14C	109.5
C6—C7—H7A	109.2	N2—C15—N3	127.9 (2)
N1—C7—H7B	109.2	N2—C15—S1	120.94 (18)
C6—C7—H7B	109.2	N3—C15—S1	111.10 (18)
H7A—C7—H7B	107.9	N3—C16—O1	119.2 (2)
N1—C8—C9	111.9 (2)	N3—C16—C17	122.8 (2)
N1—C8—H8A	109.2	O1—C16—C17	118.0 (2)
C9—C8—H8A	109.2	C18—C17—C16	115.4 (2)
N1—C8—H8B	109.2	C18—C17—H17	122.3
C9—C8—H8B	109.2	C16—C17—H17	122.3
H8A—C8—H8B	107.9	N2—C18—C17	124.9 (2)
C13—C9—C8	111.4 (3)	N2—C18—Cl2	115.60 (19)
C13—C9—C10	112.8 (3)	C17—C18—Cl2	119.48 (19)
C8—C9—C10	107.9 (3)	O1—C19—H19A	109.5
C13—C9—H9	108.2	O1—C19—H19B	109.5
C8—C9—H9	108.2	H19A—C19—H19B	109.5
C10—C9—H9	108.2	O1—C19—H19C	109.5
C11—C10—C9	111.0 (3)	H19A—C19—H19C	109.5
C11—C10—H10A	109.4	H19B—C19—H19C	109.5
C9—C10—H10A	109.4	C8—N1—C12	111.5 (2)
C11—C10—H10B	109.4	C8—N1—C7	111.9 (2)
C9—C10—H10B	109.4	C12—N1—C7	111.8 (2)
H10A—C10—H10B	108.0	C15—N2—C18	113.9 (2)
C12—C11—C10	110.0 (3)	C16—N3—C15	115.1 (2)
C12—C11—C14	110.9 (3)	C16—O1—C19	118.0 (2)
C10—C11—C14	112.3 (3)	C15—S1—C1	103.40 (12)
C12—C11—H11	107.8		
			
C6—C1—C2—C3	−1.7 (4)	N3—C16—C17—C18	−1.0 (4)
S1—C1—C2—C3	−175.51 (19)	O1—C16—C17—C18	178.0 (2)
C1—C2—C3—C4	1.2 (4)	C16—C17—C18—N2	1.2 (4)
C2—C3—C4—C5	0.0 (4)	C16—C17—C18—Cl2	−178.17 (19)
C3—C4—C5—C6	−0.7 (4)	C9—C8—N1—C12	59.6 (3)
C3—C4—C5—Cl1	178.5 (2)	C9—C8—N1—C7	−174.4 (2)
C4—C5—C6—C1	0.1 (4)	C11—C12—N1—C8	−57.7 (3)
Cl1—C5—C6—C1	−178.95 (17)	C11—C12—N1—C7	176.2 (2)
C4—C5—C6—C7	178.6 (2)	C6—C7—N1—C8	157.2 (2)
Cl1—C5—C6—C7	−0.5 (3)	C6—C7—N1—C12	−76.9 (3)
C2—C1—C6—C5	1.0 (3)	N3—C15—N2—C18	−0.1 (4)
S1—C1—C6—C5	174.74 (17)	S1—C15—N2—C18	178.03 (18)
C2—C1—C6—C7	−177.5 (2)	C17—C18—N2—C15	−0.7 (4)
S1—C1—C6—C7	−3.8 (3)	Cl2—C18—N2—C15	178.70 (18)
C5—C6—C7—N1	109.8 (3)	O1—C16—N3—C15	−178.6 (2)
C1—C6—C7—N1	−71.8 (3)	C17—C16—N3—C15	0.4 (3)
N1—C8—C9—C13	177.8 (3)	N2—C15—N3—C16	0.2 (4)
N1—C8—C9—C10	−57.8 (3)	S1—C15—N3—C16	−178.04 (17)
C13—C9—C10—C11	179.1 (3)	N3—C16—O1—C19	0.5 (3)
C8—C9—C10—C11	55.6 (4)	C17—C16—O1—C19	−178.5 (2)
C9—C10—C11—C12	−54.9 (4)	N2—C15—S1—C1	15.0 (2)
C9—C10—C11—C14	−178.9 (3)	N3—C15—S1—C1	−166.64 (18)
C10—C11—C12—N1	55.2 (4)	C2—C1—S1—C15	−95.9 (2)
C14—C11—C12—N1	180.0 (3)	C6—C1—S1—C15	90.2 (2)
